# BAK and ARG Have Moisturizing, Anti‐Inflammatory, Antioxidant, and Antioxidant Bioactivities, in the Zebrafish Model

**DOI:** 10.1111/jocd.70128

**Published:** 2025-04-02

**Authors:** Xufeng Jiang, Weiguo Wang, Yuqing Wei, Wenxuan Cheng, Shoulin Li, Linjing Du, Wenping Xu, Yang Zhang

**Affiliations:** ^1^ Shanghai Key Laboratory of Chemical Biology, School of Pharmacy East China University of Science and Technology Shanghai China; ^2^ Ugel Cosmetics Pte. Ltd. Singapore City Singapore

**Keywords:** acetyl hexapeptide‐8, bakuchiol, biological activity, skin health, zebrafish

## Abstract

**Background:**

Bakuchiol (BAK) and acetyl hexapeptide‐8 have favorable biological activities and have promising applications in the cosmetic industry. However, the evaluation of the biological activity of both alone or in combination has not been reported.

**Aims:**

The biological activity of both alone or in combination was evaluated based on the zebrafish model.

**Methods:**

Zebrafish embryos were induced to form models using different methods. Add BAK and Argireline YOUth peptide oil solution MB (ARG, containing 0.125% acetyl hexapeptide‐8) to treat zebrafish embryos, and they were evaluated for their restorative effects on preexisting damage in zebrafish embryos.

**Results:**

BAK and ARG were able to reduce water loss from the caudal fin of zebrafish induced by 0.9% NaCl solution. They were able to alleviate the UVB‐induced decrease in the expression level of the skin tightness‐related gene (*ELN*/*COL1a1b*) in zebrafish embryos, and BAK or ARG effectively reversed the increase in β‐galactosidase activity induced by exposure to H_2_O_2_ solution and restored telomerase activity in zebrafish embryos. In addition, both were able to counteract oxidative stress and mitochondrial damage in zebrafish embryos as a result of LPS treatment. Finally, BAK and ARG were also effective in suppressing the increase in neutrophil counts and inflammatory cytokine levels in zebrafish embryos due to LPS exposure. Notably, BAK and ARG were more effective when used in combination.

**Conclusions:**

Acetyl hexapeptide‐8 promotes the bioactivity of BAK in zebrafish embryos ( *Danio rerio* ). BAK and ARG have moisturizing, anti‐inflammatory, antioxidant, and antioxidant bioactivities in the zebrafish model.

**Abbreviations**
AQP3: aquaporin 3
ARG: Argireline YOUth peptide oil solution MB
BAK: bakuchiol
CAT: catalase
COL1a1b: collagen, type I, alpha 1b
DCFH‐DA: 2′,7′‐dichlorodihydrofluorescein diacetate
ELN: elastin
GSH: glutathione
LPS: lipopolysaccharide
MDA: malondialdehyde
qPCR: quantitative real time PCR
SA‐β‐Gal: senescence‐associated β‐galactosidase
SOD: superoxide dismutase
TRAP: telomeric repeat amplification protocol

## 1 Introduction

Bakuchiol (BAK), predominantly found in the leguminous plant *Psoralea corylifolia* L. [1], is a monoterpene phenol compound characterized by a chiral center [2]. BAK exhibits therapeutic pharmacological properties and is widely utilized. Notably, it has been reported to exert cardioprotective effects [3] and demonstrate anticancer activity [4]. In the cosmetic industry, BAK presents considerable application potential. In China, BAK was approved by the National Medical Products Administration (NMPA) in 2022 and is authorized for use as a cosmetic ingredient during the current monitoring period. It is reported that BAK possesses strong bioactivity, including antiaging, antioxidant, and anti‐inflammatory effects [5, 6].

Acetyl hexapeptide‐8 is an active peptide known for its antiwrinkle properties. Studies have shown that it competes with SNAP‐25 for positions in the complex, affecting the formation and stability of the SNARE complex [7]. This prevents vesicles from effectively binding to the membrane to release acetylcholine, blocking muscle electrical impulses, and ultimately inhibiting neurotransmitter release. Therefore, acetyl hexapeptide‐8 can relax facial muscles, achieving an antiwrinkle effect. However, as a water‐soluble short peptide, acetyl hexapeptide‐8 is unstable. Research indicates it has poor diffusion and a low partition coefficient, making it difficult to penetrate biological barriers and lipid membranes, which leads to low delivery efficiency and targeting [8]. Therefore, in the cosmetic industry, adding other components can enhance its stability and skin permeability.

Zebrafish are notable for their short life cycle, transparency, and significant genetic similarity to humans. Therefore, zebrafish have become an important model for pharmacological or toxicological studies [9, 10]. Currently, zebrafish have become a key model for efficacy verification in the cosmetic field. Similar to mammals, zebrafish skin is divided into two layers: the epidermis, which consists of multiple layers of keratin‐forming cells (KCs), and the dermis, which contains a mixture of fibroblasts, KCs, pigment cells, and immune cells [11, 12]. Meanwhile, zebrafish are thought to have a highly homologous skin structure [13]. In addition, 71.4% of human protein‐coding genes and 82% of disease‐causing genes have been reported to have clear zebrafish direct homologs [14].

This study evaluated the moisturizing, antiwrinkle, antiaging, antioxidant, and anti‐inflammatory effects of BAK and Argireline YOUth peptide oil solution MB (ARG, containing 0.125% acetyl hexapeptide‐8). Additionally, the study explored the differences in effects when BAK and ARG were used together versus when they were used individually.

## 2 Materials and Methods

### 2.1 Zebrafish Embryos Culture and Exposure

Zebrafish adult AB strain and *Tg(lyz: dsRed)* were purchased from China Zebrafish Resource Center (Wuhan, China), and the *Tg(Xla.Eef1a1: mlsEGFP)* strain was purchased from Nanjing YSY Biotechnology Company Ltd. Zebrafish were cultured in a light: dark ratio of 14 h: 10 h. Zebrafish adults were cultured for 1 week for spawning. Females and males were separated and kept overnight under dark conditions. The partition was removed the next day, and the embryos were collected 1 h after spawning. After 6 h of embryo incubation, unfertilized embryos were removed. BAK (> 99%, CAS: 10309‐37‐2, Sytheon Ltd., State of New Jersey, USA) and ARG (Composition: acetyl hexapeptide‐8 = 0.125% CAS: 616204‐22‐9, Cetyl Ethylhexanoate = 86.375%, Polyglyceryl‐3 Laurate = 10%, Methyl Glucose Sesquistearate = 1%, Water = 2.5%; Lipotec S.A.U., Barcelona, Spain) were solubilized using zebrafish medium and sonicated until all were dissolved. The combination of BAK and ARG is named using the code PLA‐3D. Embryos were divided into 10–30 embryos per group and exposed to BAK and ARG. Different compounds were used for exposure, including BAK (2000 ng/mL), ARG (10 ng/mL), and PLA‐3D (BAK 2000 ng/mL: ARG 5 ng/mL). The duration of exposure was 72 h, and the exposure solution was changed every 24 h. The exposure solution was used for the treatment of the patients.

### 2.2 Modeling and Treatment of Dehydration in Zebrafish Embryos

Zebrafish embryos were modeled for dehydration in zebrafish using a 0.9% NaCl solution. A control group, a model group, and the sample groups were set up. Normal zebrafish embryo medium was used for culture in the control group. Zebrafish embryo medium containing a 0.9% NaCl solution was used for culture in the model group. The sample groups were cultured using zebrafish embryo medium containing a 0.9% NaCl solution +BAK (or ARG, or PLA‐3D) solution. Zebrafish embryo tails were photographed using a microscope (SMZ745T, Nikon, Tokyo, Japan) after 72 h of exposure, and the tail area was counted using image J software (National Institutes of Health, Bethesda, MA, USA). The experiment was repeated three times for biology and three times for technology. Zebrafish embryos were 10 per group, and the experiment was repeated three times.

### 2.3 β‐Galactosidase Activity

A control group, a model group, and the sample groups were set up. Zebrafish embryo medium containing a 2 mM H_2_O_2_ solution was used for culture in the model group. The exposed completed embryos were washed three times using Zebrafish Embryo Medium. Follow the method according to the Senescence β‐galactosidase Staining Kit instructions (Beyotime, Shanghai, China). Fixation was performed using β‐galactosidase fixative for 15 min and washed three times with PBS. Add 1 mL of staining working solution and incubate overnight at 37°C. Finally, it was observed under a microscope, and photographs were taken. Zebrafish embryos were 10 per group, and the experiment was repeated three times.

### 2.4 Telomerase Activity

Zebrafish were washed three times with zebrafish‐specific culture medium and subjected to a TRAP assay based on probe‐based qPCR. Telomerase extracts were obtained by adding TRAP‐specific cell lysate to dissociate the tissue and then incubating on ice for 30 min. The samples were centrifuged (12 000 g at 4°C for 20 min) and the supernatant was collected. Total protein concentration was assessed using the BCA assay. Then, 3 μg of protein was added to the TRAP premix to 20 μL in a 96‐well plate according to the manufacturer's (Tianjingsha, Shanghai, China) instructions. The qPCR was performed using a real‐time fluorescence quantitative PCR system (QuantStudio 5, Thermo Fisher, Waltham, USA): 30 min at 30°C and 5 min at 95°C; 45 cycles of 15 s at 95°C, 15 s at 57.5°C, and 30 s at 72°C. A standard curve for telomerase activity was obtained using a 1:9 serial dilution of the positive control extract. Data are expressed in relative telomerase activity units. The experiment was repeated three times for biology and three times for technology. Primers are shown in Table 1.

**TABLE 1:** The primers sequence for telomerase.

Gene	Primer
*TERT*	Forward: CGGTATGACGGCCTATCACT
	Reverse: TAAACGGCCTCCACAGAGTT
*β‐Actin*	Forward: GATGATGAAATTGCCGCACTG
	Reverse: ACCAACCATGACCCTGATGT

### 2.5 DCFH‐DA Staining

After zebrafish embryo exposure was completed, fresh zebrafish embryo medium was used to wash three times. DCFH‐DA (MCE, Shanghai, China) staining was performed by adding 10 μM DCFH‐DA for 30 min in the dark and washed three times with fresh zebrafish embryo medium. Zebrafish embryos were fixed using low melting point agar, and images were taken using confocal microscopy (A1R, Nikon, Tokyo, Japan). The fluorescence intensity of the zebrafish embryos was analyzed using Image J. Zebrafish embryos were 10 per group, and the experiment was repeated three times.

### 2.6 JC‐1 Staining

After zebrafish embryo exposure was completed, fresh zebrafish embryo medium was used to wash three times. Add 5 μM JC‐1 (Solarbio, Beijing, China) staining under darkness for 30 min. Other methods were the same as in Section 2.4. Zebrafish embryos were 10 in each group, and the experiment was repeated three times.

### 2.7 Mitochondrial and Neutrophil Content in Zebrafish Embryos

*Tg(Xla.Eef1a1: mlsEGFP)* [15] and *Tg(lyz: dsRed)* [16] strains of zebrafish were used. Zebrafish embryos were washed three times, and zebrafish were fixed using low melting point agar. Images were captured using a confocal microscope, and fluorescence intensity was analyzed using image J. The images were analyzed using a confocal microscope. Zebrafish embryos were 10 per group, and the experiment was repeated three times.

### 2.8 qPCR

Zebrafish embryos in groups of 30 were exposed. Completed embryos were washed using fresh zebrafish embryo medium. Total RNA from zebrafish embryos was extracted using the Trizol method (Servicebio, Wuhan, China) and reverse‐transcribed into cDNA using a reverse transcription kit (Servicebio, Wuhan, China). Finally, SYBR dye (Servicebio, Wuhan, China) was added, and the mRNA levels were determined by qPCR instrumentation. The determination of the expression of *ELN* and *COL1a1b* required the use of UVB (313 nm, 15 min) to establish a zebrafish model. Different compounds were used in other test methods for induction, followed by total RNA extraction. The experiment was repeated three times for biology and three times for technology. All results were calculated by 2^−∆∆Ct^ and the relative expression of the genes was quantified. GAPDH was used as an internal reference gene. Primers are shown in Table 2.

**TABLE 2:** The primers sequence for qPCR.

Gene	Primer
*AQP3*	Forward: CAGACTTTCCAAATCCGCAAC
	Reverse: GCAAACCAAGATTCCAAGAGTAGC
*ELN*	Forward: CAGCCGACGTGCCTGAA
	Reverse: GGCCACCAATCCCAGTAGCA
*COL1a1b*	Forward: GCTTTGTGGATATTCTGCTGGC
	Reverse: ATTTGGCATGGCTCTGGTTTC
*IL‐1β*	Forward: TTCCCCAAGTGCTGCTTATT
	Reverse: AAGTTAAAACCGCTGTGGTCA
*IL‐6*	Forward: TCAACTTCTCCAGCGTGATG
	Reverse: TCTTTCCCTCTTTTCCTCCTG
*IL‐8*	Forward: GCTTCTGATCTGCACGACTG
	Reverse: CCACGTCTCGGTAGGATTGA
*TNF‐α*	Forward: ACATCAGCTGCACGTCTGAA
	Reverse: CGTGCAGATTGAGCGGATTG
*GAPDH*	Forward: GTAACTCCGCAGAAAAGCC
	Reverse: CAAAAGAAACTAACACACAC

### 2.9 Statistical Analysis

Three independent experiments were analyzed using SPSS version 27.0 statistical software (IBM Co. Ltd., NY, USA). The results were expressed as mean ± standard deviation (SD), and one‐way analysis of variance (ANOVA) was employed to analyze the difference among groups. Dunnett's test and Tukey's test were performed to analyze the significance of each treatment and control group. Values of *p* < 0.05 were considered statistically significant. Lowercase letters (a, b, c, d, e) were used to mark differences between groups.

## 3 Results

### 3.1 Analysis of Moisturizing Efficacy of BAK and ARG Based on Zebrafish Model

**FIGURE 1:**
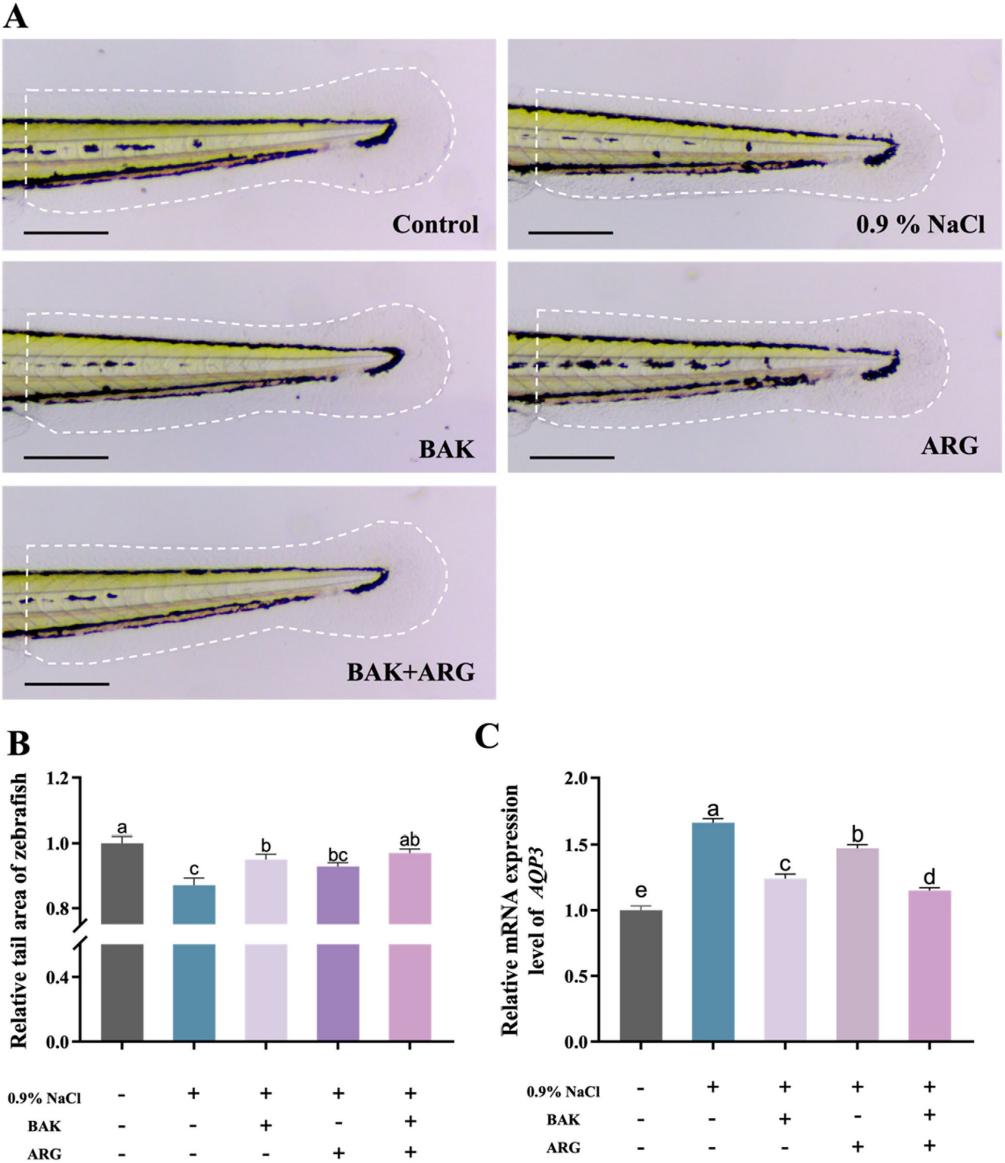
Effect of BAK and ARG on the area of the tail of zebrafish embryos. (A) BAK and AGR restore 0.9% NaCl‐induced water loss in the caudal fin of zebrafish embryos. The scale: 100 μm. (B) Area of the tail of zebrafish embryos. (C) Expression of *AQP3* mRNA. Error bars represent mean ± SD for three replicates. Lowercase letters indicate significant differences: *A, b, c, d = p < 0.05*.

Tail water loss was induced in zebrafish embryos using a 0.9% NaCl solution. The 0.9% NaCl solution‐induced a significant reduction in the tail area of zebrafish embryos compared to the control group. BAK or ARG was able to restore the tail area of zebrafish embryos induced by the 0.9% NaCl solution (Figure 1A). It is interesting to note that this restoration was even more effective when BAK and ARG (2000: 5) were added together (Figure 1B). In addition, the expression of AQP3 was analyzed (Figure 1C). The results showed that BAK or ARG was able to reduce the increase in AQP3 expression in zebrafish embryos induced by the 0.9% NaCl solution. Similarly, the simultaneous addition of BAK and ARG resulted in a more significant decrease in AQP3 expression.

### 3.2 Analysis of the Firming Efficacy of BAK and ARG Based on Zebrafish Models

**FIGURE 2:**
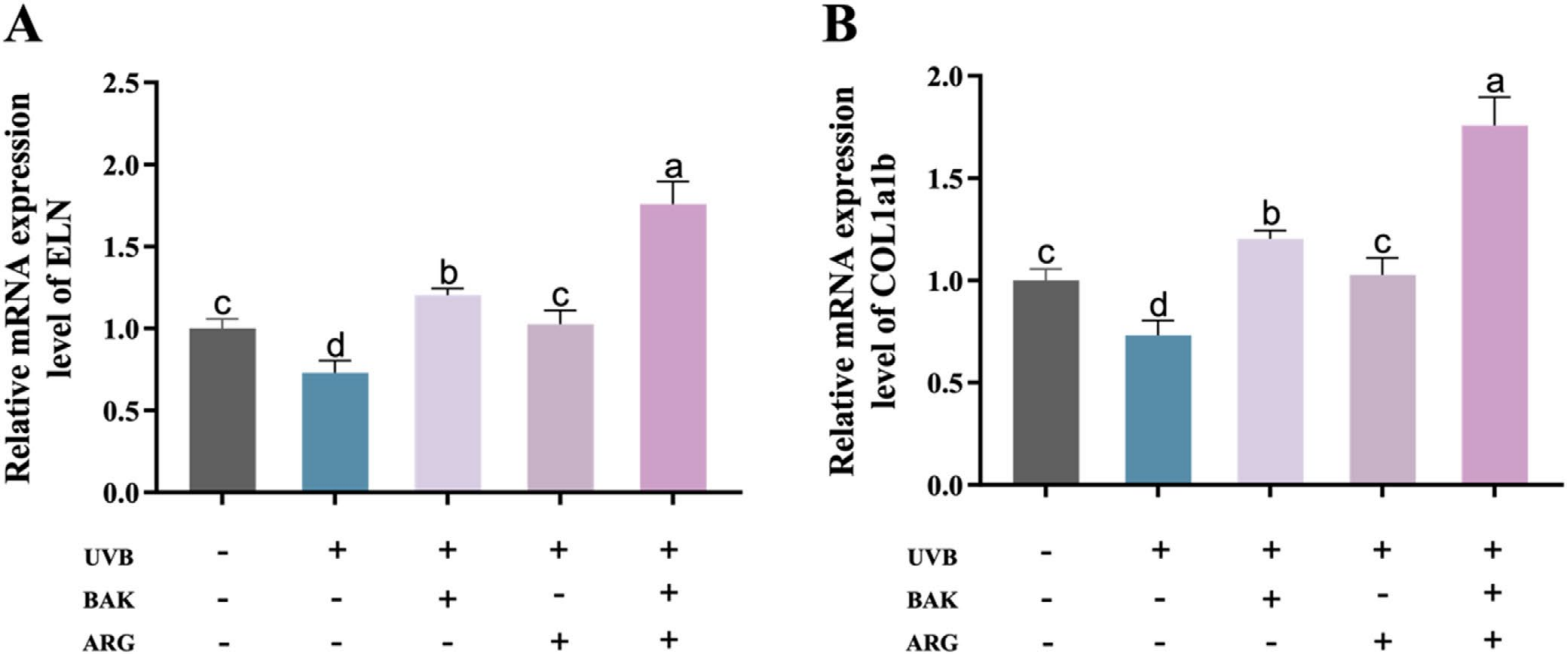
Effects of BAK and ARG on the expression of *ELN* and *COL1a1b* mRNA in zebrafish embryos. (A) Expression of *ELN* mRNA. (B) Expression of *COL1a1b* mRNA. Error bars represent mean ± SD for three replicates. Lowercase letters indicate significant differences: *A, b, c, d = p < 0.05*.

Epidermal damage was induced in zebrafish embryos using UVB. UVB‐induced a significant reduction in the expression of *ELN* and *COL1a1b* mRNA in zebrafish embryos compared to the control group (Figure 2A,B). BAK or ARG was able to restore the expression levels of *ELN* and *COL1a1b* mRNA in zebrafish embryos induced by UVB. Similarly, the simultaneous addition of BAK and ARG resulted in a more significant decrease in *ELN* and *COL1a1b* expression.

### 3.3 Analysis of Antioxidant Efficacy of BAK and ARG Based on Zebrafish Modeling

**FIGURE 3:**
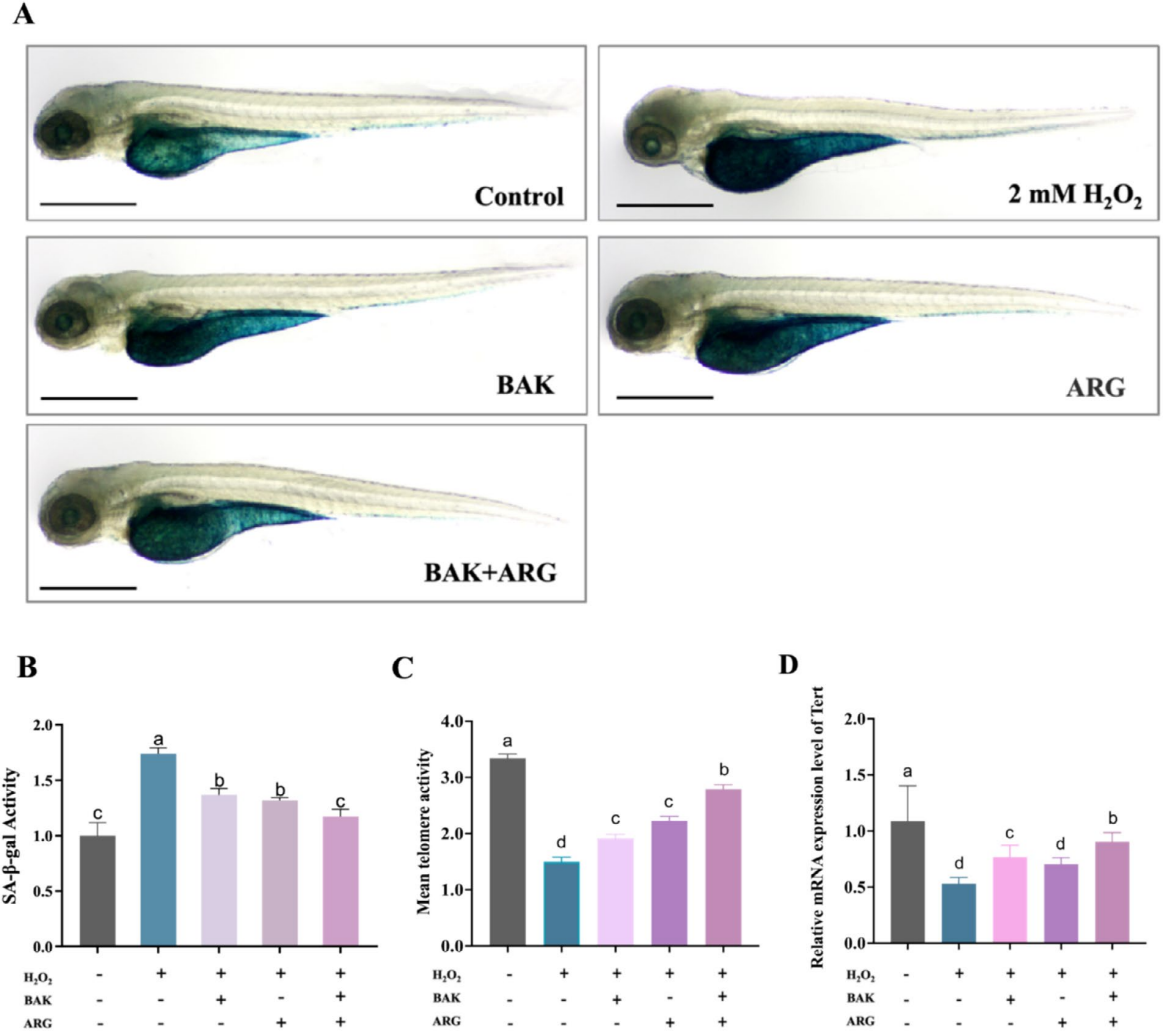
Effect of BAK and ARG on β‐galactosidase in zebrafish embryos. The scale: 200 μm. (A) β‐galactosidase staining of zebrafish embryos. (B) Viability of SA‐β‐Gal in zebrafish larvae. (C) Mean telomere activity. (D) Relative mRNA expression level of tert. Error bars represent mean ± SD for three replicates. Lowercase letters indicate significant differences: *A, b, c, d = p < 0.05*.

Zebrafish embryos were induced to cause oxidative damage using H_2_O_2_ solution. Compared with the control group, SA‐β‐Gal activity was significantly elevated in H_2_O_2_‐induced zebrafish embryos (Figure 3A,B). SA‐β‐Gal activity levels in H_2_O_2_‐induced zebrafish embryos were restored by BAK or ARG. Telomerase activity was significantly reduced in H_2_O_2_‐induced zebrafish embryos compared with control (Figure 3C,D). BAK or ARG restored telomerase activity in H_2_O_2_‐induced zebrafish embryos. This restoration was better when both BAK and ARG were added.

### 3.4 BAK and ARG Restore LPS‐Induced Oxidative Stress Levels in Zebrafish Embryos

**FIGURE 4:**
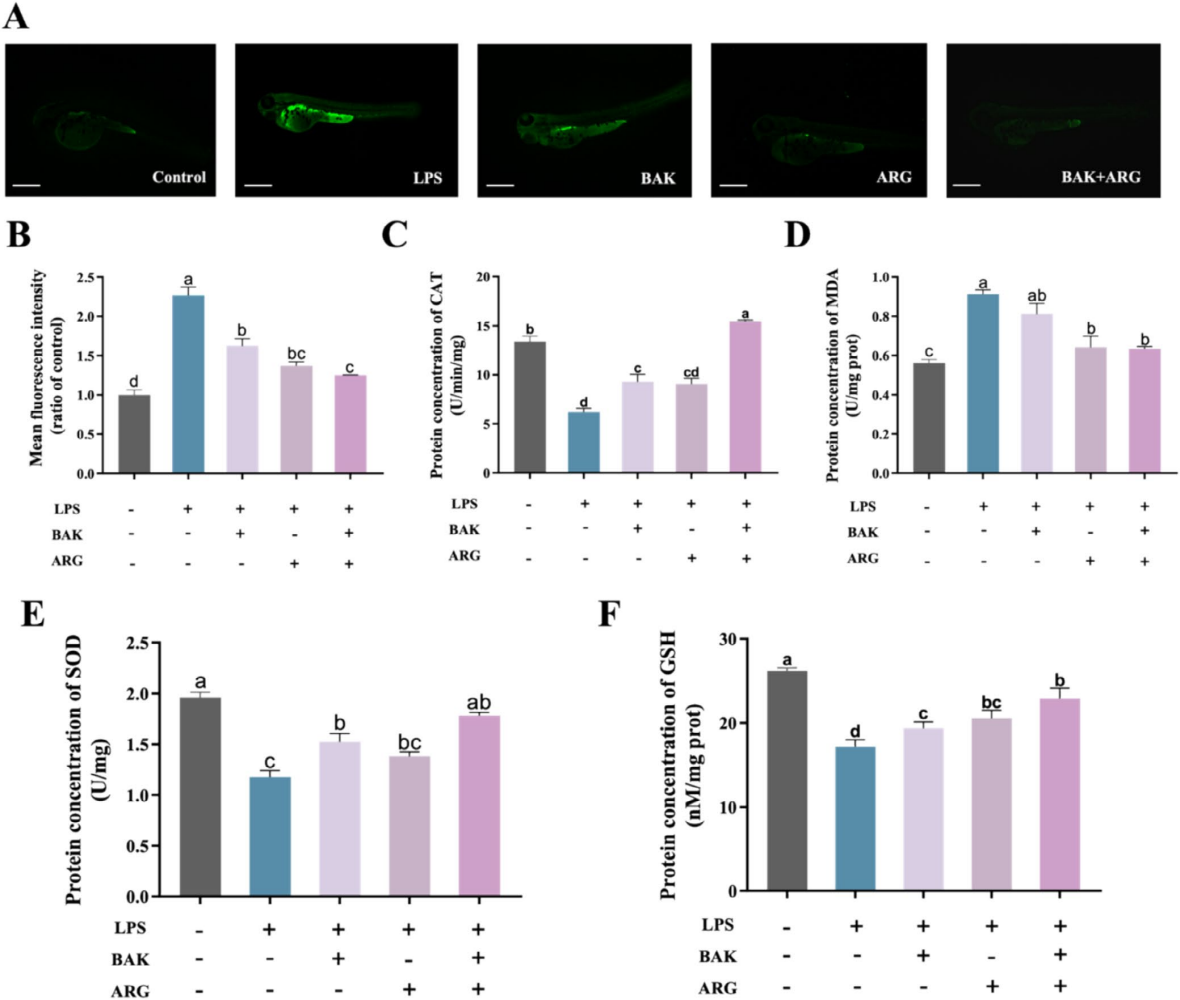
Effects of BAK and ARG on oxidative stress in zebrafish embryos. The scale: 200 μm. (A) DCFH‐DA staining of zebrafish embryos. (B) Fluorescence intensity of DCFH‐DA in zebrafish embryos. (C) CAT activity. (D) MDA content. (E) SOD activity. (F) GSH activity. Error bars represent mean ± SD for three replicates. Lowercase letters indicate significant differences: *A, b, c, d = p < 0.05*.

Oxidative stress was induced in zebrafish embryos using LPS. LPS‐induced a significant increase in ROS levels (Figure 4A,B) and MDA content (Figure 4D), as well as a significant decrease in CAT (Figure 4C), SOD (Figure 4E), and GSH (Figure 4F) activity in zebrafish embryos compared to the control group. BAK or ARG were able to restore the levels of ROS, MDA content, and related oxidative kinase activities in LPS‐induced zebrafish embryos. Similarly, this recovery was better when BAK and ARG were added simultaneously.

### 3.5 BAK and ARG Restore LPS‐Induced Mitochondrial Damage in Zebrafish Embryos

**FIGURE 5:**
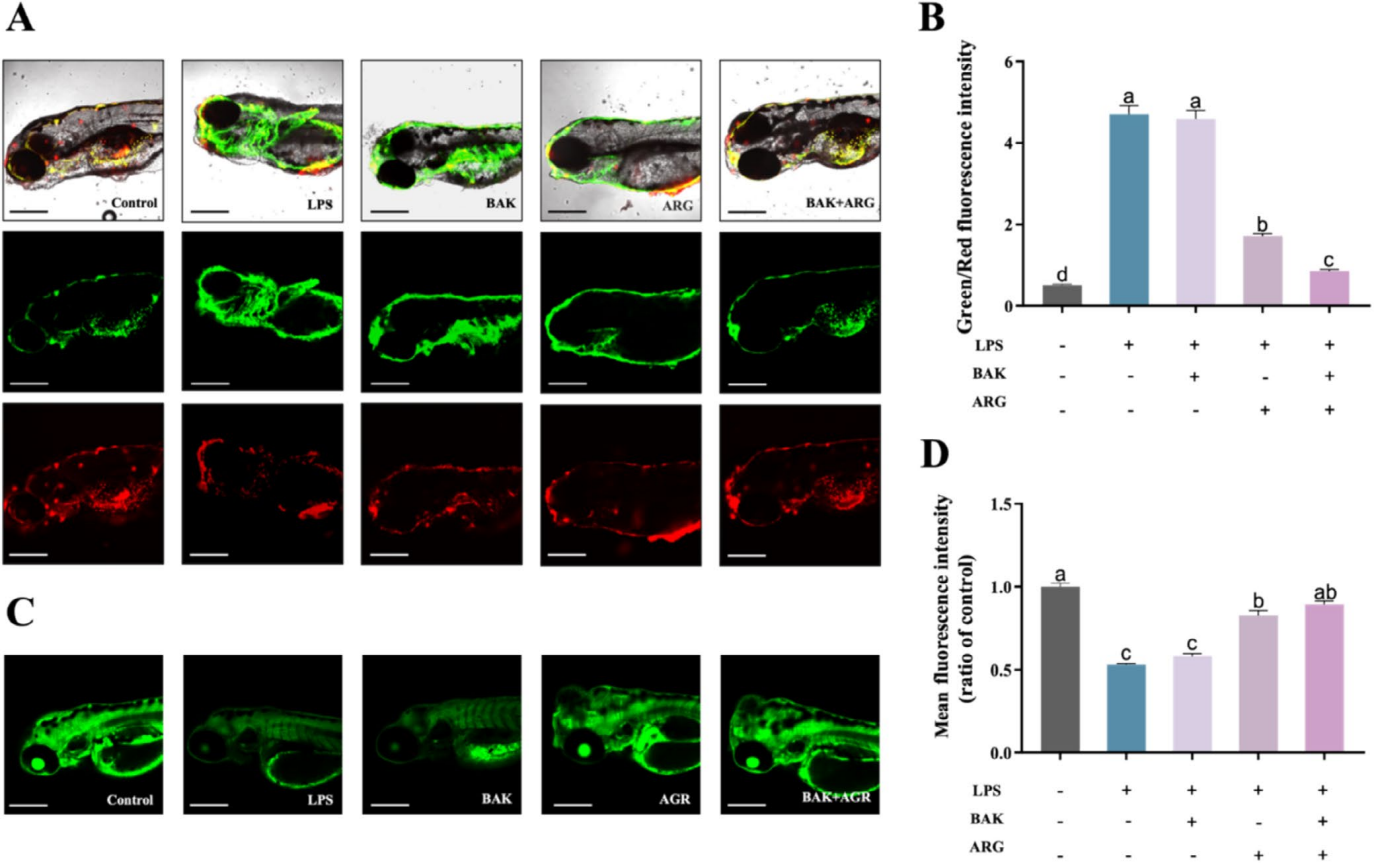
Effects of BAK and ARG on mitochondria in zebrafish embryos. (A) JC‐1 staining of zebrafish embryos. The scale: 100 μm. (B) Ratio of green/red color in the results of JC‐1 staining. (C) Fluorescence intensity of mitochondria in zebrafish. The scale: 100 μm. (D) Mean fluorescence intensity of *Tg(Xla.Eef1a1: MlsEGFP)* straining. Error bars represent mean ± SD for three replicates. Lowercase letters indicate significant differences: *A, b, c, d = p < 0.05*.

Mitochondrial damage was induced in zebrafish embryos using LPS. LPS‐induced altered mitochondrial membrane potential and reduced mitochondrial number in zebrafish embryos compared to controls. BAK or ARG were able to restore mitochondrial membrane potential (Figure 5A,B) and mitochondrial number (Figure 5C,D) in LPS‐induced zebrafish embryos. Similarly, this recovery was better when BAK and ARG were added simultaneously.

### 3.6 BAK and ARG Restore LPS‐Induced Neutrophil Infiltration and Elevated Inflammatory Factor Expression in Zebrafish Embryos

**FIGURE 6:**
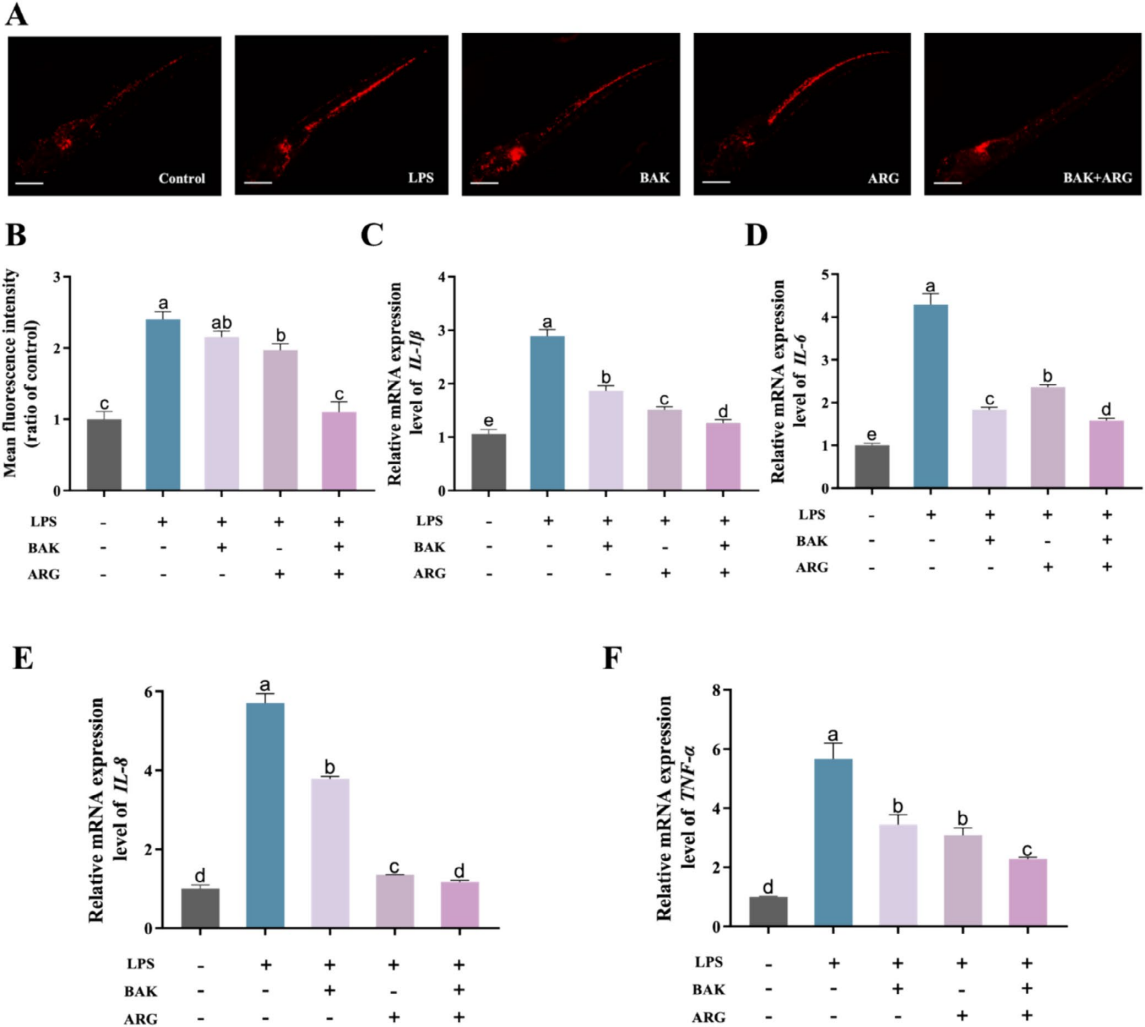
Effects of BAK and ARG on the number of neutrophils and expression of inflammatory factors in zebrafish embryos. (A) Neutrophils in zebrafish embryos in vivo. The scale: 200 μm. (B) Mean fluorescence intensity of *Tg(lyz: DsRed)* zebrafish. (C) Expression of *IL‐1β* mRNA. (D) Expression of *IL‐6* mRNA. (E) Expression of *IL‐8* mRNA. (C) Expression of *TNF‐α* mRNA. Error bars represent mean ± SD for three replicates. Lowercase letters indicate significant differences: *A, b, c, d = p < 0.05*.

Induction of zebrafish embryos using LPS produced neutrophil infiltration and altered expression of inflammatory factors. LPS‐induced zebrafish embryos showed significantly higher neutrophil numbers (Figure 6A,B) and increased expression of inflammatory factors compared to controls. BAK or ARG were able to restore LPS‐induced alterations in neutrophil numbers in zebrafish embryos. BAK or ARG were able to restore LPS‐induced alterations in *IL‐1β* (Figure 6C), *IL‐6* (Figure 6D), *IL‐8* (Figure 6E), and *TNF‐β* (Figure 6F) in zebrafish embryos. The results of this study show that LPS‐induced neutrophil damage and altered expression of inflammatory factors in zebrafish embryos were significantly higher in the zebrafish embryos. BAK or ARG can effectively ameliorate LPS‐induced inflammatory injury in zebrafish embryos. Similarly, this restoration was even more effective when both BAK and ARG were added.

## 4 Discussion

Bakuchiol and acetyl hexapeptide‐8 are widely utilized in the cosmetic industry due to their remarkable bioactivities. While numerous studies have examined these compounds individually, there is a notable lack of research on their synergistic effects. This study investigates the bioactivities of BAK, ARG, and a combination of BAK + ARG. Given the instability and low permeability associated with pure acetyl hexapeptide‐8, ARG was selected as an alternative. The objective was to ascertain whether the incorporation of ARG could enhance the bioactivity of BAK.

Zebrafish serve as an excellent model organism, capable of developing various injury models through methods such as 0.9% NaCl induction, UVB irradiation [17], H_2_O_2_ induction [18], and LPS induction [19]. Dehydration of zebrafish embryos can be induced using a 0.9% NaCl solution, leading to a reduction in tail fin area. Both BAK and ARG were effective in reversing the dehydration of zebrafish embryos' tail fins, indicating their potential moisturizing properties. AQP3, an important aquaporin, plays a critical role in hydration [20]. Further research demonstrated that BAK and ARG could restore AQP3 expression levels in zebrafish larvae. The combination of these compounds showed a more pronounced effect on the restoration of tail fin area and reduction in AQP3 expression compared to either substance alone.

ELN and COL1a1b are essential proteins associated with skin firmness [21, 22]. Exposure to UVB radiation damages the epidermis of zebrafish, leading to a reduction in the expression levels of *ELN* and *COL1a1b*. The study demonstrated that both BAK and ARG could alleviate this decrease in expression. β‐Galactosidase serves as a significant marker for aging‐related damage [23]. Induction by H_2_O_2_ accelerates oxidative damage and aging in zebrafish embryos. The findings indicated that both BAK and ARG were effective in reducing the increase in β‐galactosidase activity, thereby suggesting their potential roles in promoting skin firmness and combating aging. Furthermore, both compounds were found to restore telomerase activity, indicating additional antiaging potential. When administered together, the effects on enhancing *ELN*/*COL1a1b* expression and diminishing β‐galactosidase activity were more pronounced than those observed with individual treatments.

Oxidative stress serves as a critical indicator of cellular damage [24]. Key markers such as ROS and associated enzymes were assessed, including SOD, CAT, GSH, and MDA. The results indicated that LPS induction led to an increase in ROS levels while simultaneously reducing MDA content in zebrafish embryos. Additionally, there was a notable decrease in the activity of SOD, CAT, and GSH enzymes. The administration of BAK or ARG effectively normalized oxidative stress levels toward those observed in the control group. Furthermore, the combined treatment resulted in oxidative stress levels aligning more closely with those of the control group, thereby demonstrating the antioxidant potential of these compounds.

Mitochondria are essential for providing the energy necessary for life processes and play a vital role in maintaining organismal health. This study also investigated the capacity of BAK and ARG to alleviate mitochondrial damage. It was found that LPS induction increased mitochondrial membrane potential while decreasing mitochondrial quantity within zebrafish embryos. Treatment with either BAK or ARG restored these parameters to control levels, with combined treatment yielding even more favorable outcomes.

Upon inflammatory challenge, zebrafish exhibit a significant increase in neutrophil counts to combat inflammation. The study demonstrated that LPS induction resulted in a substantial rise in neutrophil numbers within zebrafish embryos. Treatment with BAK or ARG led to a reduction in neutrophil levels, bringing them closer to control values. Furthermore, the effects of BAK and ARG on the expression of inflammatory cytokines were investigated. The results indicated that both BAK and ARG effectively diminished the elevated expression of inflammatory cytokines induced by LPS in zebrafish embryos. Consequently, these compounds exhibit potential anti‐inflammatory bioactivity. Notably, the combination treatment proved more effective in alleviating LPS‐induced inflammatory damage in zebrafish embryos.

## 5 Conclusion

The current study used a zebrafish model to explore the bioactivities of BAK and ARG, which included moisturizing, antiwrinkle, antiaging, antioxidant, and anti‐inflammatory properties. BAK and ARG were able to 0.9% NaCl‐induced, UVB irradiation, H_2_O_2_ solution‐induced, and LPS‐induced skin damage in zebrafish. Succinctly, BAK and ARG were able to promote zebrafish caudal fin growth, promote the expression of antiwrinkle genes (*ENL* and *COL1a1b*), reduce the level of β‐galactosidase activity, increase telomerase activity, reduce the level of oxidative stress, and reduce inflammation‐induced neutrophilia and inflammatory factor expression. Most importantly, the results suggest that the addition of ARG promotes the bioactivity of BAK.

## Author Contributions

X.J., W.W., Y.W., W.C., S.L., and L.D. performed the research. X.J. and W.W. designed the research study. W.X. and Y.Z. contributed essential reagents or tools. Y.W., W.C., S.L., and L.D. analyzed the data. X.J., W.W., and Y.Z. wrote the paper.

## Conflicts of Interest

The authors declare no conflicts of interest.

## Data Availability

The data that support the findings of this study are available from the corresponding author upon reasonable request.
